# Editorial: The Clinical and Ethical Practice of Neuromodulation – Deep Brain Stimulation and Beyond

**DOI:** 10.3389/fnint.2017.00032

**Published:** 2017-11-09

**Authors:** Markus Christen, Sabine Müller

**Affiliations:** ^1^Institute of Biomedical Ethics and History of Medicine, University of Zurich, Zurich, Switzerland; ^2^Department of Psychiatry and Psychotherapy, Mind and Brain Research, Charité - Universitätsmedizin Berlin, Berlin, Germany

**Keywords:** deep brain stimulation, ethics, medical, neuromodulation, social issues, neurology, psychiatry

Neuromodulation is among the fastest-growing areas of medicine, involving many diverse specialties and affecting hundreds of thousands of patients with numerous disorders worldwide. It can briefly be described as the science of how electrical, chemical, and mechanical interventions can modulate the nervous system function. A prominent example of neuromodulation is deep brain stimulation (DBS), an intervention that reflects a fundamental shift in the understanding of neurological and psychiatric diseases: namely as resulting from a dysfunctional activity pattern in a defined neuronal network that can be normalized by targeted stimulation. The application of DBS has grown remarkably and more than 130,000 patients worldwide have obtained a DBS intervention in the past 30 years—most of them for treating movement disorders. These numbers will grow further for several reasons. First, DBS is investigated for various novel neurological and psychiatric indications. Second, current research suggests that stimulation may be more beneficial if it is applied earlier in the course of the disease, especially for Parkinsonian patients. Third, the number of countries, centers, and companies that get involved in this field is steadily increasing.

This Frontiers Research Topics provides an overview on the current discussion beyond basic research in DBS and other brain stimulation technologies. Researchers from clinical disciplines (e.g., neurology, neurosurgery, and psychiatry), neuroethics, social science, law, and economics who are working on broader clinical and social issues related to DBS and related neuromodulation technologies have contributed to this research topic. In the following, we provide a brief overview on the content of the e-book on “The clinical and ethical practice of neuromodulation – deep brain stimulation and beyond.”

The paper from Ineichen and Christen exemplifies the impressive publication activity in the field. They analyzed more than 7,000 papers published between 1991 and 2014 on DBS using quantitative methods. The study confirms known trends within the field such as the emergence of psychiatric indications with a particular focus on depression and the increasing discussion of complex side-effects such as personality changes. Other findings are more surprising, e.g., that hardware-related issues are far more robustly connected to ethical issues compared to impulsivity, concrete side-effects or death/suicide. This indicates that the bioethical discussion on DBS may underestimate ethical problems due to DBS hardware.

The issue of complex side-effects are in the center of the opinion article of Cyron. He argues that psychiatric side effects are an integral part of DBS for Parkinson's disease. Particularly, hypomania, reckless behavior, suicidality, changes in personality, and moral competence are issues with a confounding ethical impact. He pleads for a sober and unprejudiced discussion about neuropsychiatric effects of DBS for Parkinson's disease.

Kocabicak et al. address the vividly discussed question whether there is still need for microelectrode recording, given that the subthalamic nucleus can be well-visualized with MR imaging. Based on the literature and their own experience, they argue that intra-operative electrophysiology is not necessary to find the STN. However, they have decided not to abandon it, particularly because with multiple-electrode recordings, an alternative trajectory is immediately available, if necessary.

Müller et al. broaden the view on psychiatric neurosurgery in their contribution. They compare the rivaling paradigms DBS and modern ablative procedures with microsurgery or radiosurgery, and argue that none of the procedures is absolutely superior. Rather, they have different profiles with respect to advantages and disadvantages. They conclude, that the patients' social situation, individual preferences, and individual attitudes are crucial when deciding, which of the methods are preferable.

Crowell et al. describe their observations of the response of patients with treatment-resistant depression to DBS. They find that the typical time course consists of different phases, beginning with changes in mood reactivity, followed by a transient worsening prior to stabilization of response. The authors hypothesize that this characteristic recovery curve reflects the timeline of neuroplasticity in response to DBS.

Beeker et al. discuss the ethical concern that DBS for patients with major depression might threaten their ability to make autonomous decisions. The authors argue that DBS in these patients might increase the patients' autonomy by reducing anhedonia and increasing energy, so that it can rather restore than threaten autonomy.

A summary of the current state of the discussion on the many facets of DBS is provided by the “Proceedings of the Fourth Annual Deep Brain Stimulation Think Tank” from Deeb et al. that took place in 2016 and gathered leading researchers such as James Giordano, Helen Mayberg, Jens Volkmann, and Michael Okun. The spectrum of addressed topics is very large and includes research and clinical practice, policy issues such as the formation of registries and novel technological innovations such as closed-loop DBS. Readers gain an up-to-date overview when consulting this contribution.

The contribution of Glannon provides a more philosophical focus on the issue of neuromodulation. He pleads for a non-reductive materialist model of the mind and brain relation. He argues that the fact that DBS can modulate dysfunctional brain circuits to make them amenable to cognitive-behavioral therapy underscores the complimentary of brain-based and mind-based techniques in controlling the symptoms of psychiatric disorders.

Finally, Cabrera and Reiner have investigated the public's understanding of the proposed use of a non-invasive neuromodulation technique: transcranial direct current stimulation (tDCS). They investigated the use of tDCS for enhancement by analyzing and comparing online comments in key popular press articles from two different periods: pre-commercialization and post-commercialization. They found that the public's attitude has shifted from misunderstanding to cautionary realism—probably a common pattern when analyzing the public reception of new neurotechnologies.

Overall, the contributions that form this eBook on clinical and ethical practice of neuromodulation demonstrate the many facets of a fascinating new research field that poses important and challenging ethical and social questions. It is encouraging to see that researchers from many different disciplines have begun to tackle them.

## Author contributions

All authors listed have made a substantial, direct and intellectual contribution to the work, and approved it for publication.

### Conflict of interest statement

The authors declare that the research was conducted in the absence of any commercial or financial relationships that could be construed as a potential conflict of interest. The reviewer ARB and handling Editor declared their shared affiliation.

